# Do the Various Indirect Bonding Techniques Provide the Same Accuracy for Orthodontic Bracket Placement? (Randomized Clinical Trial)

**DOI:** 10.1155/2024/5455197

**Published:** 2024-01-22

**Authors:** Ammar Sh. Al-Ubaydi, Dheaa Al-Groosh

**Affiliations:** ^1^College of Dentistry, University of Baghdad, Baghdad, Iraq; ^2^Ministry of Health, Baghdad, Iraq; ^3^Orthodontic Department, College of Dentistry, University of Baghdad, Baghdad, Iraq

## Abstract

**Background:**

For orthodontic treatment to be effective, bracket placement must be precise to make the finishing stage easier, leading to an ideal occlusion with minimal intervention. This study aimed to evaluate the accuracy of manual and digital bracket positioning techniques utilizing computer-aided design and computer-aided manufacturing (CAD/CAM) jigs, 3D-printed indirect bonding trays (IBT), and double-layer vacuum-formed thermoplastic IBT.

**Methods:**

This study was done by scanning the dental arch of 30 orthodontic patients. The virtual setup and bracket positioning were performed with the Insignia™ system for ten patients, and 3D Maestro® software was used for the virtual setup of the remaining 20 patients. At the same time, the bracket positioning of 10 patients was done digitally by the 3D Maestro® software and the remaining 10 patients manually through the Ray Set® device. IBT were fabricated by CAD/CAM system, 3D printer, and vacuum-formed thermoplastic machine. A virtual bracket position was compared to the actual bracket position using the best-fit method of 3D digital superimposition in Geomagic® Control X™ (CX) software to determine how accurate it was in terms of linear and angular accuracy. Statistical analyses using SPSS 26.0 including Bland–Altman plots were used to assess the intra-examiner reproducibility. Shapiro–Wilk test was used to measure normality distribution. Wilcoxon matched-pairs signed rank test was used to analyze the differences between bracket positions within each group.

**Results:**

Although there were obvious positional discrepancies between several readings, they were still within clinically acceptable ranges.

**Conclusions:**

All types of IBT would translate the planned position of the bracket from the digital and manual techniques to the teeth of patients with accepted precision in both linear and angular measurements; in addition, the error rate is about the same for all types of IBT. This trial is registered with NCT05549089.

## 1. Introduction

One of the most important steps in orthodontic therapy is the placement and bonding of the brackets, which substantially impact the clinical result [1–4]. Using direct or indirect bonding methods, the brackets can be attached to the teeth surfaces[5]. Since saliva is present and some teeth are inaccessible, direct bonding frequently requires more chairside time and is less precise [6]. To improve the precision of bracket placement, Silverman et al. [7] proposed indirect bonding, which became a common choice [1]. Several studies found that indirect virtual bonding facilitated accurate bracket positioning compared to direct vision or with loupes direct bonding in the linear and angular measurements [8, 9]. The indirect bonding technique reduces bracket placement errors in angulation, vertical, and horizontal positions [10], and significantly saves chairside time, while it requires high costs related to its laboratory work [11] and needs well-trained orthodontists to accurately transfer the estimated bracket position to the teeth, especially on dentitions with varied malocclusion and intraoral disorders [12, 13].

Concerning the design and fabrication, transfer trays for indirect bonding techniques (IBT) have been improved, such as polyvinyl siloxane type [14, 15], vacuum-formed type [16–18] combinations of polyvinyl siloxane and vacuum-formed types [19], 3D-printed type [20–22], and customized transfer jigs [23]. The conventional approach, in which brackets and tubes are bonded in their ideal positions manually, generates a double-layer IBT that utilizes 1/2 mm soft clear mouthguard material to hold the brackets with a 1-/1.5-mm-hard clear splint biocryl stabilizing layer over it. It is less expensive, requires more laboratory time, and is subject to human mistakes [18]. On the other hand, modern 3D printing and computer-aided design and computer-aided manufacturing (CAD/CAM) technologies have been introduced in the orthodontic field, where virtual bonding is done digitally. For 3D-printed IBT, resin printer creates trays by organizing layers of liquid resin on top of each other; while for CAD/CM jigs, custom milling of a transparent rigid thermoplastic material (polymethyl methacrylate) is done to create individual jig that hold the bracket and fit the occlusal surfaces of the teeth [24]. They offer several benefits, like accurate 3D images, easy storage of files, and precision in image processing and outcome estimation [25].

The indirect bonding method consists of two phases: the laboratory and clinical phases [6]. Brackets are bonded in the lab on the patient's orthodontic study model before being transferred to the clinical part using a special transfer tray [25, 26].

In recent years, the development of CAD/CAM technologies specifically for indirect bonding systems such as Orapix (Orapix Co., Ltd., Seoul, Korea), Insignia™ (Ormco Corporation, Glendora, California, United States), and Orthocad (Cadent Inc., Carlstadt, New Jersey, United States) designed a digital model using a CAD/CAM application and fabricate a transfer jig using these procedures, which improve bonding between the bracket and the tooth [27–29].

This prospective study aimed to assess manual and digital bracket positioning accuracy utilizing CAD/CAM jig, 3D-printed and double-layer vacuum-formed thermoplastic IBT.

## 2. Methods

### 2.1. Trial Design

This study is a prospective randomized controlled clinical trial registered in ClinicalTrials.gov (NCT05549089) and approved by the research and ethics committee of the dental college at Baghdad University (number 624422/2021). CONSORT 2010 flow diagram and CONSORT 2010 checklist [30] were included.

### 2.2. Participants, Eligibility Criteria, and Settings

A total of 30 participants in orthodontic treatment were enrolled in the study. All participants had permanent dentitions with complete clinical crown heights classified as CL I malocclusion with mild to moderate crowding (little index ≤6 mm); the treatment plan is without extraction. The exclusion criteria are large restored teeth, defective teeth, and insufficient interdental space, which causes limitation during bracket placement.

Patients recruited at the orthodontic department of the dental college of Baghdad University and at a private dental clinic from May 2022 to February 2023; all patients and their parental guidance received informed consent.

**Figure 1:**
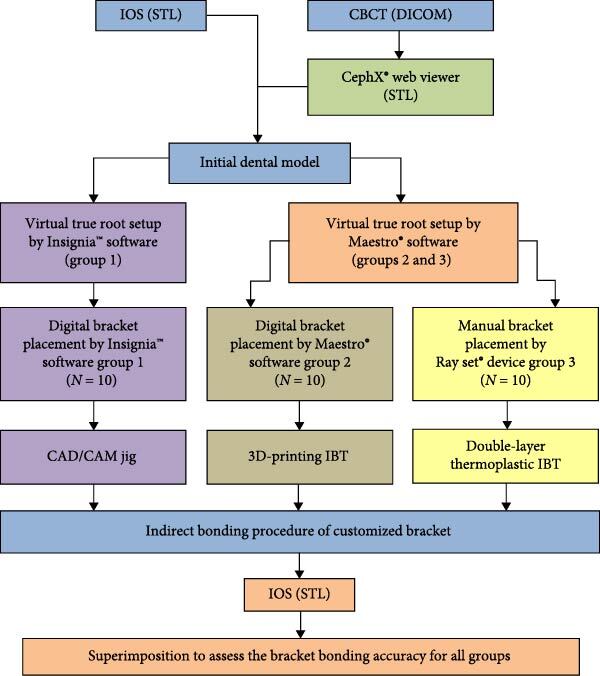
Schematic diagram of study flow.

The patients were divided into three groups and the study design is shown in Figure 1:

Group 1: Digital bracket placement by Insignia™ system (*N* = 10).

Group 2: Digital bracket placement by Maestro® software (*N* = 10).

Group 3: Manual bracket placement by Ray set® device (*N* = 10).

An intraoral scanner (3Shape TRIOS 3, Copenhagen, Denmark) used to produce a digital model of each patient.

### 2.3. Sample Size Calculation

According to previous research [16, 28], the sample size of teeth was determined. With an observed sample size of *n* = 240 teeth in each group (24 teeth in each patient measured from right to left 1st molars in both arches), power analysis for the Wilcoxon matched-pairs signed rank test (two-tailed) using *G* ^*∗*^ Power 3.1.9.4 shows 85% power to detect a small effect size (Cohen's *d* = 0.4) at the 0.05 significant level.

### 2.4. Randomization and Allocation Concealment

From 93 participants assessed for eligibility, only 30 patients suitable for inclusion criteria were enrolled following a random protocol to ensure good collaboration. Patients were randomly assigned per practice, 10 in each practice, to one of the three treatment groups via a block randomization procedure with a block size of six, using a computer-generated list of random numbers. The allocation sequence was concealed from the researcher and the patient using identical, sequentially numbered, opaque, sealed envelopes. The randomization and allocation procedures were performed at the trial center. When a patient was determined to be eligible, the researcher explained the trial and the patient gave signed informed consent to participate. Then, the researcher opened the envelope, and the patient was assigned to a treatment group. In the first group, patients were treated with the Insignia™ system (Ormco, Orange, United States); in the second group, they were treated with digitally customized brackets (Maestro® software); and in the third group, treatment was done with the manually customized brackets (Ray set® device).

### 2.5. Blinding

Since both the researcher and the patient would have to know which therapy group they were in, only the statistician was blinded.

## 3. Methodology

### 3.1. Insignia™ System Group

**Figure 2:**
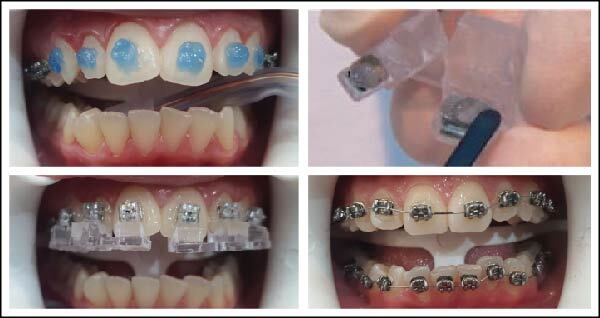
Insignia® bonding procedures.

Production of CAD/CAM jigs and indirect bonding procedure:

The digital model produced by intraoral scanner (IOS) sent to the Ormco® (Insignia™ TruRoot® system). The Insignia approval software consists of five stages:

1st Step—Preview: *treatment preferences*. 2nd Step—Evaluation and modification: *expansion & posterior torque*, *smile arc*, *incisor torque and anteroposterior movement*. 3rd Step—Verify proper alignment: *torque, then vertical; rotations, then tip; in-out, then mesio-distal*. 4th Step—Evaluate occlusion and occlusal contacts. 5th Step—Appliance check: *bracket placement*, *bracket torque*, *archwires and jig groupings*. Finally, twin brackets with customized bases, bonding jigs for indirect bonding and archwires were manufactured by Ormco® and used as specified by the Insignia™ TruRoot® system. Then, the transfer jigs with their brackets were placed intraorally and bonded on the tooth surface according to the following protocol: start to control saliva secretion by placing super absorbent pads from the buccal side of the upper molars and the buccal and lingual sides of the lower molars. The retractor for the lips and cheeks is positioned in such a way as to allow a clear view of the whole oral cavity, including the buccal surfaces of the molars. The facial surfaces of the teeth were etched with an etching gel for 15 s, sprayed with water for at least 5 s, and then dried with air spray. Then, a small amount of adhesive was spread on the back of each bracket base; 3M Transbond™ XT light-curable adhesive was used. The jigs with cotton tweezers were grasped and rolled from the lingual cusp or incisal edge to the facial surface to avoid disrupting the adhesive layer by moving the bracket base along the tooth surface. Once firmly seated, pressure on the jigs was maintained with finger force (applied 45° to the enamel surface). This procedure ensures uniform contact between each pad and the respective tooth [31]. If adhesive extruded between the tooth and pad, a microbrush was dipped in the bonding agent to remove the excess. Each bracket underwent a 5-s-light cure so that the curing light was used for half the specified time, then released the finger pressure and completed the second half of the curing process passively (Figure 2).

### 3.2. Maestro® Software Group

#### 3.2.1. Production of 3D-Printed IBT and Indirect Bonding Procedure

The digital model produced by IOS was uploaded into the Maestro 3D Ortho Studio (Age Solutions S.r.l., Pisa, Italy) to produce an aligned digital model with virtual location of the brackets. The virtual bonding was used to attach preadjusted brackets (Roth, 0.022-inch slot, Discovery, Dentaurum, Ispringen, Germany).

**Figure 3:**
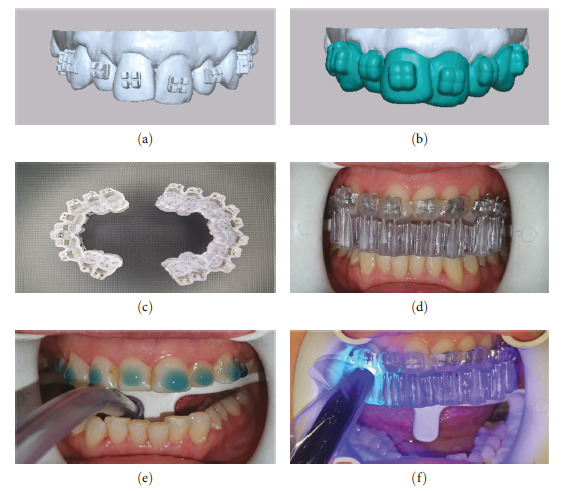
(a) Digital models with brackets in STL format. (b) Indirect bonding trays (IBT) in STL format. (c) 3D-printed IBT with brackets. (d) Prebonding intraoral check for the placement and fitness of IBT. (e) and (f) Bonding procedure.

Digital models with brackets were exported in STL format (Figure 3(a)). The IBT were developed using the virtual model (Figure 3(b)) and then sent to a 3D printer (Asiga max U.V., Dental Direkt GmbH, Germany) using biocompatible IBT resin (Detax GmbH, Germany) (Figure 3(c)).

Then the 3D printer IBT with its bracket placed intraorally and bonded on the tooth surface using 3M Transbond™ XT light-curable adhesive (Figure 3(d)–3(f)). After removing the 3D-printed trays from the mouth, residual adhesives were removed from around the brackets.

### 3.3. Ray Set® Device Group

#### 3.3.1. Production of Double-Layer IBT and Indirect Bonding Procedure

The digital model produced by IOS was uploaded into the Maestro 3D Ortho Studio (Age Solutions S.r.l., Pisa, Italy) to produce a digital aligned model but without brackets. The digitized models were exported in STL format and sent to a 3D printer to create the well-aligned dental model by TEC resin (Detax GmbH, Germany). Then the dental model was placed on the Ray set device® (Biaggini medical device, Italy) [32] (https://www.biaggini.it), which is a 3D goniometer control system, which measure each tooth's three dimensions (tip, torque, and rotation). The preadjusted brackets (Roth, 0.022-inch slot, Discovery, Dentaurum, Ispringen, Germany) were manually bonded on the plaster patients' initial model by ortho adhesive (Transbond XT, 3M Unitek, St. Paul, Minn) according to the prescription of printed aligned model that measured on Ray set device®.

The silicone-based impression material was used to take a precise impression of patients' initial models. Plaster casts were produced from the silicone molds following a 24-hr crystallization period at room temperature. Then, a thin layer of separating agents (Metrodent® denture separating medium, U.K.) was applied to the tooth surfaces of the cast [33].

**Figure 4:**
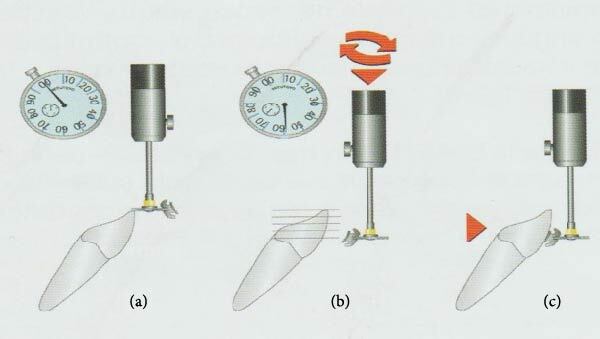
Bracket positioning procedure on the Ray set device®. (a) Detection of the occlusal reference point, (b) leveling of the slot height with the FA point, and (c) moving the bracket toward the tooth surface.

**Figure 5:**
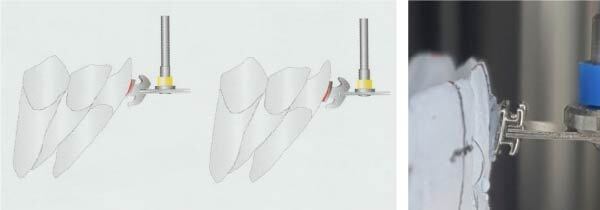
Bracket bonding procedure on the Ray set device®.

The bonding step was done using the Ray set device® according to the measurements acquired from the true root virtual digital setup printed model, as follows:

- The bracket was fitted firmly to the holder's flexible plier, which is the one extremity of the Ray is set device® bracket holder. While the holder's free side on the other extremity was lied on the occlusal reference point. The vertical gauge was set to zero when the bracket holder touched the occlusal reference point (Figure 4(a)). The bracket lowered to the definitive slot height, the facial axis (FA) point (Figure 4(b)). This point previously marked on the crown is now hidden by the bracket (Figure 4(c)).
- The bonding agent was applied (3M Transbond™ XT light-curable adhesives). After that, the bracket holder rotated until the bracket was in the correct contact position for bonding. So the bonding agent that relines the bracket base will correct eventual anatomical discrepancies and add torque values where necessary. Finally, the light curing of the adhesive material is about 5 s (Figure 5).

### 3.4. Fabrication of IBT

**Figure 6:**
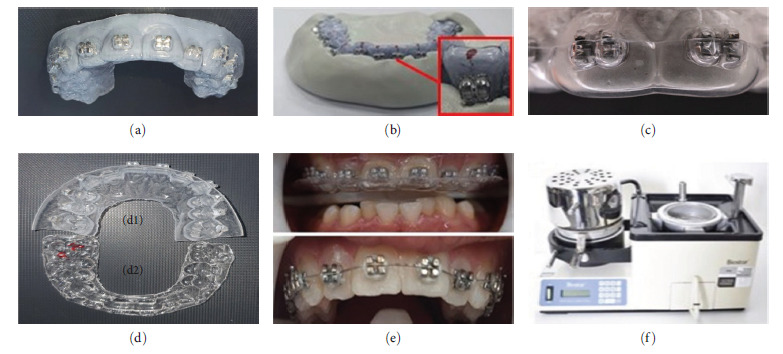
Fabrication of double-layer IBT. (a) Forming the soft sheet material by pressing it over brackets on the plaster cast. (b) Heavy body silicon material used to close the undercuts from the base of the cast until it reached gingival one-third of the brackets. (c) Trimming the outer layer's excess until it covers occlusal two-thirds of the brackets. (d) Layers of Thermo-former IBT, d1: 1 mm inner layer (soft film); and d2: 1 mm outer layer (hard film). (e) Intraoral bonding. (f) Vacuum forming machine.

Double-layer guide plates were manufactured by 3A MEDES® Thermo-former with a 1-mm inner layer (soft film) and 1-mm outer layer (hard film). A soft sheet material was pulled over the working model using a BioStar VI vacuum forming machine (Scheu-Dental GmbH, Iserlohn, Germany) and left to cool. The tray was then detached with the attachments in place. After that, the cast was covered with a heavy body silicon material to close the undercuts from the base of the cast until it reached to gingival one-third of the brackets. Then, press the hard layer to cover the occlusal two-thirds of the brackets. Finally, the two layers were removed from the dental cast, trimmed, cleaned with a toothbrush and brackets were placed in the double-layer IBT. The customized composite bracket base was sandblasted with aluminium oxide (50 *µ*m) and finally cleaned with Alcohol. Then the double-layer IBT with its bracket placed intraorally and bonded on the tooth surface using 3M Transbond™ XT light-curable adhesive (Figure 6).

### 3.5. Evaluation of Bracket Placement Accuracy

**Figure 7:**
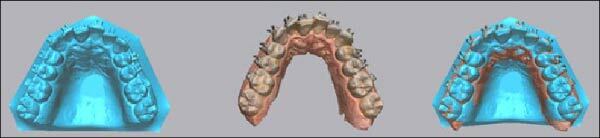
3D digital superimposition (best-fit method) data. The combination between the simulative dentition bracket (blue color) and intraoral scan data of the posttransfer dentition bracket.

**Figure 8:**
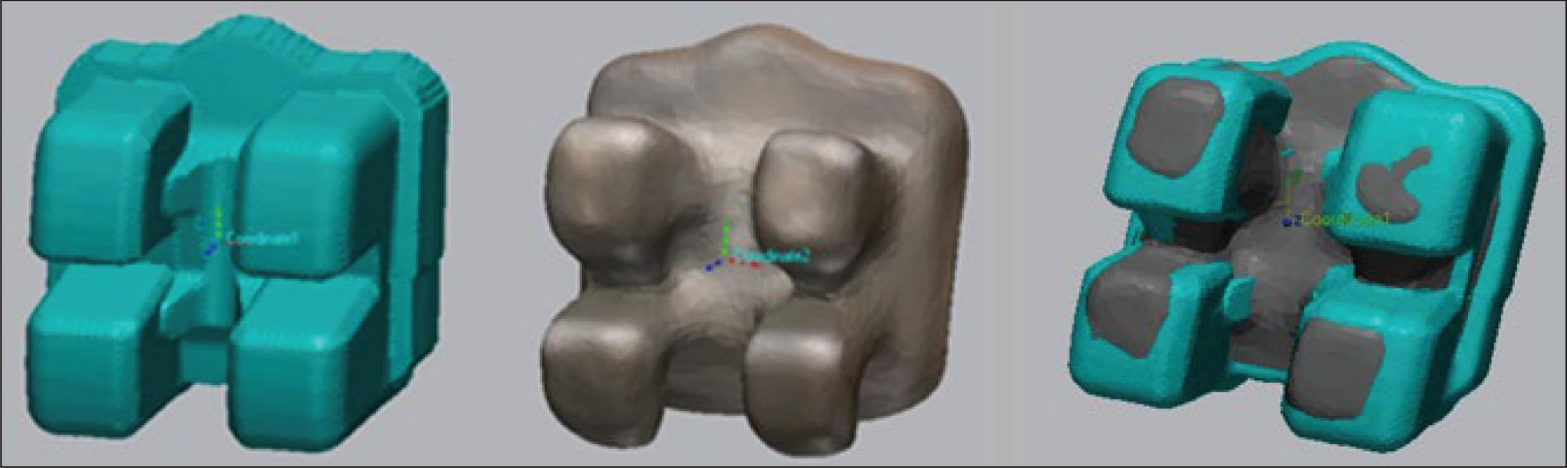
Separating the brackets from the buccal teeth surfaces and creating the coordinate system on both simulative (green color) and postoperative (gray color) brackets, then comparing the linear and angular measurements between the two coordinate systems.

After bracket bonding, the teeth were completely dried and sprayed with an antireflective material using a scanning preparation spray (Dentaco GmbH & Co. K.G. in Germany), then scanned and converted to digital format using an intraoral scanner (3Shape TRIOS), then Geomagic® Control X™ (CX) software (version 2020.1.1; 3D Systems Inc, Rock Hill, SC) used to register each bracket on the virtual to the actual model as follows:

- The virtual and actual brackets were imported into CX software and superimposed between them through best-fit alignment (Figure 7).
- Each virtual and actual bracket was separated from the buccal tooth surface following its outlines. Then, each bracket was automatically designed with a local coordinate system in its midpoint. So, the coordinate system was placed at the same point on the virtual and actual brackets (Figure 8).

Linear and angular deviations for each virtual and actual brackets coordinate system were calculated automatically regarding the original coordinate system.

**Figure 9:**
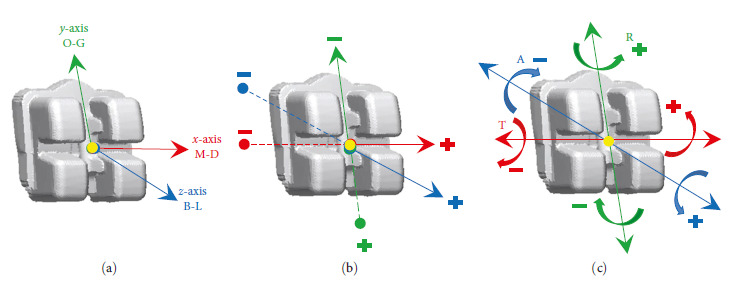
Twin bracket with 22 slot. (a) 3D-coordinate system. (b) Linear deviation of the bracket. (c) Angular deviation of the bracket.

According to Park et al. [34] define the linear and angular deviation of the coordinate system as shown in Table 1 and Figure 9.

### 3.6. Statistical Analysis

Statistical analyses were carried out using SPSS 26.0 (SPSS Inc, Chicago). Bland–Altman plots were used for intra-examiner reproducibility. The descriptive statistic includes mean and standard deviation for the liner and angular deviations. According to the results of the Shapiro–Wilk test, the variables did not have a normal distribution. For nonnormally distributed variables, the Wilcoxon matched-pairs signed rank test was used to compare the deviations with a clinically acceptable range for the measurement values was assumed if it was within a linear displacement of 0.5 mm, and an angular discrepancy of 2°, according to the American Board of Orthodontics objective grading system (ABO-OGS) [35]. The significance level was established at *P*  < 0.05. Also, the frequency of directional error and bracket bonding failure at removing the three types of IBT was calculated for each tooth.

## 4. Results

The demographic data of the study participants were 22.71 ± 5.23 years old, 73.3% female and 26.7% male, Caucasian Iraqi adults with a good socioeconomic level and educational background.

In this study, 720 brackets (60 dental arches, 30 patients) were bonded; 240 brackets (20 dental arches, 10 patients) using the Insignia jigs, 240 brackets (20 dental arches, 10 patients) using the 3D-printed IBT and 240 brackets (20 dental arches, 10 patients) using the double-layer vacuum-formed thermoplastic IBT.

**Figure 10:**
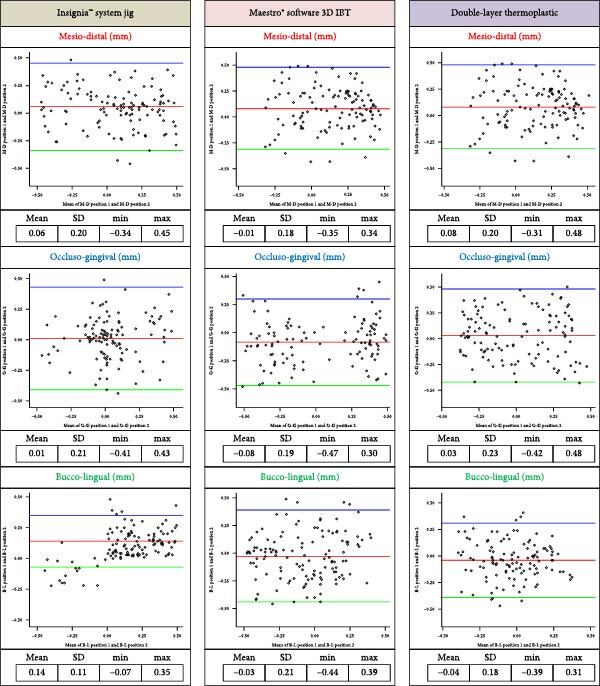
Translation (linear) difference between two interval times of 120 brackets randomly selected for each group (Bland–Altman plot).

**Figure 11:**
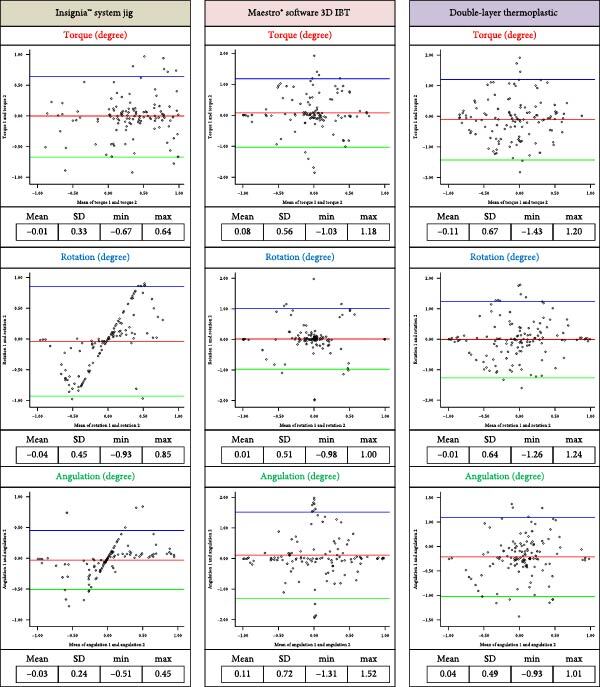
Orientation (angular) difference between two interval times of 120 brackets randomly selected for each group (Bland–Altman plot).

Reproducibility assessments were reported in plots shown in Figures 10 and 11. In which Bland–Altman analyses of agreement between measurements of individual bracket positions performed at two time points. The difference between the original and repeat measurements (mm or degrees) is plotted against the mean of the original and repeat measurements for each bracket pair. Each circle represents one bracket pair. 95% of measurement differences lie between the limits indicated by the top and bottom dashed lines. The Insignia™ jigs group have revealed that the mean differences of linear deviation ranging from 0.01 to 0.14 mm, and from −0.01° to −0.04° for angular deviation. While, the 3D printing IBT group revealed that the mean differences of linear deviation ranging from −0.01 to −0.08 mm, and from 0.01° to 0.11° for angular deviation. However, the double-layer vacuum-formed thermoplastic IBT group revealed that mean differences of linear deviation ranging from −0.04 to 0.08 mm, and from −0.11° to 0.04° for angular deviation. The Bland–Altman plots showed that most data fell within a mean range of ±1.96 (SD), confirming the reliability of measuring linear and angular deviation.

Tables 2–7 showed linear and angular deviation between the virtual and actual brackets. In the Insignia™ jig group, the mesio-distal (M-D) brackets' positional deviations, occluso-gingival (O-G), and bucco-lingual (B-L) directions were less than 0.5 mm for all brackets, with a mean value (SD) of −0.06 (0.07), 0.01 (0.06), and 0.11 (0.04) mm, respectively. The angular deviations in torque, rotation, and angulation of the bracket were less than 2° for all brackets, with a mean value of 0.04° (0.12°), −0.01° (0.09°), and −0.03° (0.08°), respectively. However, in the 3D printing IBT group, the MD brackets' linear deviations, O-G, and B-L directions were less than 0.5 mm for all brackets, with a mean value (SD) of −0.02 (0.10), −0.13 (0.11), and −0.02 (0.07) mm, respectively. The angular deviations in torque, rotation, and angulation of the bracket were less than 2° for all brackets, with a mean value of 0.09° (0.20°), 0.01° (0.16°), and 0.11° (0.18°), respectively. On the other hand, in the double-layer vacuum-formed thermoplastic IBT group, the linear deviations of brackets in the MD, O-G, and B-L directions were less than 0.5 mm for all brackets, with a mean value (SD) of 0.09 (0.09), 0.03 (0.13), and −0.04 (0.05) mm, respectively. The angular deviations in torque, rotation, and angulation of the bracket were less than 2° for all brackets, with a mean value of −0.10° (0.18°), 0.02° (0.26°), and 0.07° (0.16°), respectively.

**Figure 12:**
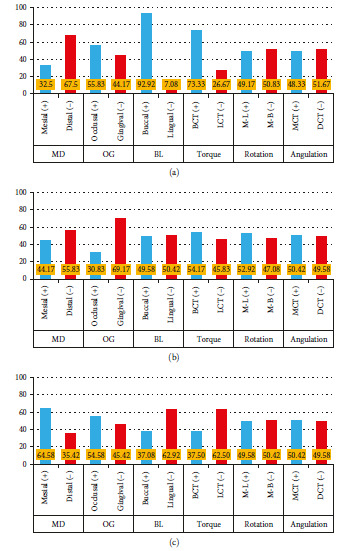
(a–c) The frequencies of directional error resulting from different IBT methods. M-D: mesio- distal (mm); O-G: occluso-gingival (mm); B-L: bucco-lingual (mm); torque (°); BCT: buccal crown torque; LCT: lingual crown torque; rotation (°); M-L: mesio-lingual; M-B: mesio-buccal; angulation (°); MCT: mesial crown tip; DCT: distal crown tip. (a) CAD/CAM Jig by Insignia™ system; (b) 3D printed IBT by Maestro® software; (c) double-layer thermoplastic IBT.

Figure 12 shows the frequencies of directional error resulting from the bracket transfer method for each tooth. In the Insignia™ jig group, the directional error was higher for the B-L direction (92.92%); while in the 3D printing IBT group, it was higher for O-G direction (69.17%). However, the double-layer vacuum-formed thermoplastic group was higher in the M-D direction (64.58%). At the same time, all the brackets were within a clinically acceptable range (0.5 mm in linear or 2° in angular) [35] in the other measurements.

The result showed nonsignificant differences between virtual and actual bonded bracket positions in all Insignia™ jigs group measurements. But when comparing the discrepancy between 3D-printed and double-layer vacuum-formed thermoplastic indirect bonding methods, in some measurements, we showed significant differences between virtual bracket positions and actual bonded bracket positions. In 3D-printed IBT, *P* values < 0.05 in five linear measurements (one in O-G and four in B-L) (Table 3) and one angular measurement (Torque) (Table 4). Whereas in double-layer vacuum-formed thermoplastic IBT, *P* values < 0.05 in nine linear measurements (three in M-D, two in O-G and four in B-L) (Table 5) and two angular measurements (one torque and one angulation) (Table 6).

Also, the result demonstrates the linear and angular deviation were measured within a mean error between −0.01 and 0.01 mm and −0.01° to −0.05°, respectively, for Insignia™ jigs. For 3D-printed IBT, the linear measurements mean error between −0.02 and −0.08 mm, and the angular measurements mean error between 0.01° and 0.11°. In comparison, the double-layer vacuum-formed thermoplastic IBT group was shown the mean error of measuring linear deviation ranging from −0.04 to 0.08 mm and from −0.11° to 0.04° for angular measurements.

The frequencies of bond failure during the removal of IBT in all groups showed an increased frequency of debonding rate in the double-layer vacuum-formed thermoplastic IBT group that was 5.00%, which is approximately two times greater than the Insignia™ jig and 3D-printed IBT that was 2.08% (Table 8).

## 5. Discussion

Accuracy of bracket placement can only be obtained with the IBT and precise intraoperative control. So, objective and accurate measurement is required. With the development of digital technology, it has been suggested that 2D assessment using digital photography is less accurate than 3D assessment using the scanned model [16, 36–39]. Our study employed an automated 3D measurement with Geomagic Control X (version 2020.1.1) to determine bonded brackets' positional and angular deviations.

In double-layer vacuum-formed thermoplastic IBT, we showed the difference between upper and lower teeth in a given linear dimension; in most tooth types, the lower arch had a larger B-L inaccuracy than the upper arch, suggesting a greater transfer accuracy of the upper trays in the B-L dimension; this can be as a result of uneven pressure used to put the lower trays, or, less likely, a systematic error in the amount of bond material applied to lower brackets. So, the operator may face more difficulties with placing the trays on the lower arch than on the upper arch due to the thickness of the trays, access, and moisture control concerns [40]. However, the efficacy of such IBTs may be questioned in the absence of precise control and accurate prediction of potential problems.

Even though the findings of linear and angular dimensional variations for all brackets in the three groups were good since they were within the acceptable clinical range, but the frequency of linear and angular errors cannot be dismissed. These errors may arise due to manufacturing defects, the presence of impurities or soft tissue impingement, the different thicknesses of the adhesives, the restricted ability to grip the IBT in the buccal area, or lopsided finger pressure on the IBT [37, 41]. For the Insignia™ jigs, the B-L translation in most brackets was more buccally positioned in most teeth (92.92%). This finding agrees with the studies reported by Paolo et al. [42] and Grunheid et al. [37]; they found that most of the brackets are placed more buccally from the buccolingual aspect when compared three different CAD/CAM indirect bonding systems in the former study, while in the second study was used the vinyl polysiloxane trays that have some flexibility, which may allow the adhesive to push the brackets a little bit more buccally. At the same time, the frequency of angular errors was in the torque for most brackets with more buccal crown torque (73.33%). This error can be attributed to the consistency of the bonding material under the customized metal bracket base [43, 44]. In addition, the jig could not cover the undercut of a bracket, and the elasticity of the separator material that would be used to make a free gap between the transfer jig and the bracket may have an effect [42]. So, these properties of the jig material could influence the accuracy of the bracket position. For the 3D-printed IBT, the O-G translation in most brackets was more gingivally positioned in all teeth (69.17%), while in the M-D aspect was more distally positioned for most brackets (55.83%). One assumption is that the 3D-printed IBT has some flexibility, so with unequal excessive finger pressure on the occlusal surface of the tray, this might displace the involved bracket more gingivally and distally on the dentition. For the double-layer vacuum-formed thermoplastic IBT method, the linear and angular error frequencies for the buccolingual aspect and torque were varied. So, most of the upper and lower teeth were displaced more lingually and had more lingual crown torque. The reason behind this is that the double-layer vacuum-formed thermoplastic IBT consists of soft and hard layers, so during insertion of the hard tray over the soft layer may push the bracket a little bit more lingually.

Other possible reasons that influence the position of the bracket when using IBT are the morphological variations in tooth structure and the bonding variation between virtual and actual bonding [45]. The amount of bond material was not standardized but rather left to operator judgment, which could have introduced an error in bracket positioning. Using precoated brackets could be one way to circumvent this problem [46].

**Figure 13:**
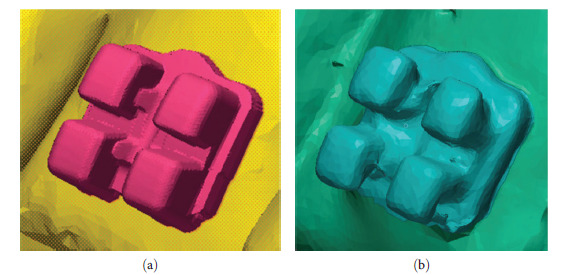
Digital model quality in this study. Example of the same bracket. (a) Virtual prebonding model with chosen bracket from bracket library. (b) Postbonding scan by 3 Shape TRIOS 3 scanner and using intra-oral scanning preparation spray.

As mentioned, this study showed that all linear and angular measurements were within the accepted clinical limit. Those results differed from the study of Bachour et al. [47], which showed acceptable linear measurements but unacceptable angular measurements compared with the clinical limits. They explained that apparent multiplanar surfaces on the digital postbonding scans could lead to image distortion due to the scattered light reflection from the metal brackets in the mouth during scanning with the iTero scanner, which operates according to the principle of light emission. This distortion on the reflective bracket surfaces will negatively affect the coordinate orientations in space and, consequentially, the angular measurements [47]. Chaudhary et al. [40] and Xue et al. [12] used a TRIOS intraoral scanner and sprayed the teeth to decrease the reflectivity of the brackets before scanning, possibly resulting in a clearer scan. Those studies' results support the idea that this study's postbonding scans with brackets were accurate enough for close superimposition on tooth structure leading to more accurate linear and angular measurements, as shown in Figure 13.

Also, Bachour et al. [47] suggested that it is possible, however, that the poor angular transfer accuracy was not entirely due to scan error but rather to an inherent property of the tray or bonding procedure that resulted in poor angular control. In addition, the multiple in vitro experiments utilizing 3D-printed trays [48], 3D-printed jigs, [49], and conventional transfer trays [16] also revealed greater linear transfer accuracy than angular transfer accuracy. For example, Niu et al. [48] found the mean M-D, B-L, and O-G transfer errors of 3D-printed trays to be 0.07, 0.13, and 0.19 mm, respectively, with errors falling within the acceptable range in 100% (M-D), 95.4% (B-L), and 96.3% (OG) of cases.

Bracket failure during indirect bonding significantly impacts the clinical efficiency of fixed orthodontic treatment [12]. Bond failures were almost invariably a result of bracket engulfment in the tray material. Niu et al. [48] explained the high transfer accuracy of linear dimensions due to using the relatively rigid printed tray material. On the other hand, they explained the low angular transfer error due to inconsistencies in the amount of bonding resin adhesive used and the tray design that provided relief for hooks and undercuts, potentially weakening angular control of bracket positioning. So to overcome this drawback, using thicker or stiffer closed trays could contribute to improved angular control. However, this would most certainly lead to increased difficulty in removing the trays postbonding, patient discomfort, and increased likelihood of bond failures. In our study, the bond failure rate during the removal of Insignia™ jigs, 3D-printed IBT, and double-layer vacuum-formed thermoplastic IBT was 2.92%, 2.08%, and 5.00%, respectively; the present study's finding was consistent with that reported in the literature for IBT in vivo [6]. In almost every incidence of bond failure in this study, the hook or gingival tie wings were covered by tray material, necessitating substantial force to remove the tray. Trimming the trays to a shorter length and reducing their thickness could reduce the bond failure rate, but at the expense of transfer precision due to the trays' diminished stiffness.

When comparing the current results to another in vivo study evaluating 3D-printed transfer trays, Chaudhary et al. [40] found low magnitudes and rates of in vivo transfer errors in the linear dimensions when using 3D-printed transfer trays (means ranging from 0.002 to 0.032 mm for M-D, 0.046–0.078 mm for O-G, and 0.000–0.016 mm for B-L dimensions). They report that 100% of positional discrepancies were within the acceptable range for the linear dimensions. Also, the greatest transfer accuracy was measured in the angular dimensions, with 100% of brackets falling within the relatively stringent acceptability range of 1° for torque, tip, and rotation. These findings are consistent with those of the present study.

Undoubtedly, digital orthodontics is the way of the future. As offices increasingly embrace 3D technologies, direct bonding may soon become an item of the past. Digital indirect bonding offers many advantages, is already being utilized in some clinics today, and will likely continue to increase in popularity as in-house 3D printing becomes more prevalent [21].

## 6. Conclusions

Accepted positional accuracy in linear and angular dimensions is achieved during bracket transfer from the digital setup to the patient's dentition via indirect bonding with Insignia™ jigs, 3D-printed IBT and double-layer vacuum-formed thermoplastic IBT. It can be said that the incidence of bracket transfer errors is roughly equivalent across all IBT kinds.

Double-layer vacuum-formed thermoplastic IBT showed the difference between upper and lower teeth in a given linear dimension and a higher bond failure rate during the removal of the transfer tray.

## Figures and Tables

**Table 1:** Directionality of bonding error.

Dimension	Measure type	Positive (+)	Negative (−)
M-D	Linear (mm): along *x*-axis	Mesial translation	Distal translation
O-G	Linear (mm): along *y*-axis	Occlusal translation	Gingival translation
B-L	Linear (mm): along *z*-axis	Buccal translation	Lingual translation
Torque	Angular (degrees): around *x*-axis	Buccal crown torque	Lingual crown torque
Rotation	Angular (degrees): around *y*-axis	Mesial-in	Mesial-out
Angulation	Angular (degrees): around *z*-axis	Mesial crown tip	Distal crown tip

**Table 2:** The difference of linear deviation between virtual and actual bracket positions of the Insignia™ jig group.

Tooth number	Mesio-distal (0.5 mm)			Occluso-gingival (0.5 mm)			Bucco-lingual (0.5 mm)		
	Mean	SD	*P* value	Mean	SD	*P* value	Mean	SD	*P* value
Maxilla									
1	−0.07	0.21	0.54	−0.13	0.14	0.89	0.07	0.24	0.17
2	−0.17	0.32	0.59	0.03	0.13	0.65	0.09	0.16	0.31
3	−0.02	0.11	0.47	0.03	0.15	0.33	0.11	0.09	0.91
4	−0.07	0.23	0.39	0.08	0.22	0.68	0.06	0.14	0.51
5	−0.07	0.19	0.2	0.04	0.2	0.91	0.11	0.11	0.54
6	−0.1	0.21	0.09	−0.02	0.15	0.95	0.21	0.19	0.65
Mandible									
1	0.10	0.14	0.32	−0.03	0.18	0.26	0.05	0.13	0.96
2	−0.04	0.16	0.44	0.12	0.16	0.84	0.08	0.11	0.17
3	−0.09	0.21	0.17	0.00	0.22	0.33	0.12	0.12	0.94
4	−0.10	0.16	0.11	0.00	0.14	0.88	0.13	0.08	0.54
5	−0.01	0.16	0.8	0.02	0.13	0.24	0.10	0.05	0.72
6	−0.05	0.14	0.08	−0.04	0.17	0.09	0.14	0.16	0.48

**Table 3:** Difference of angular orientation between virtual and actual bracket position of the Insignia™ jig group.

Tooth number	Torque (2°)			Rotation (2°)			Angulation (2°)		
	Mean	SD	*P* value	Mean	SD	*P* value	Mean	SD	*P* value
Maxilla									
1	−0.12	0.53	0.20	0.05	0.42	0.44	−0.06	0.23	0.65
2	−0.03	0.29	0.59	0.02	0.54	0.51	−0.04	0.29	0.17
3	−0.13	0.3	0.95	−0.04	0.59	0.45	0.00	0.23	0.45
4	0.05	0.32	0.37	−0.21	0.42	0.54	−0.16	0.19	0.80
5	0.02	0.24	0.47	−0.02	0.38	0.29	−0.04	0.25	0.28
6	0.1	0.4	0.24	−0.01	0.47	0.45	−0.03	0.24	0.68
Mandible									
1	−0.02	0.08	0.16	0.15	0.52	0.80	−0.01	0.18	0.96
2	−0.03	0.07	0.52	0.01	0.33	0.72	−0.07	0.16	0.72
3	0.27	0.33	0.14	0.08	0.53	0.26	0.09	0.36	0.17
4	0.11	0.42	0.96	−0.10	0.35	0.86	0.05	0.14	0.86
5	0.06	0.22	0.96	−0.01	0.50	0.17	0.05	0.32	0.88
6	0.22	0.38	0.86	−0.08	0.50	0.39	−0.17	0.24	0.29

**Table 4:** The difference of linear deviation between virtual and actual bracket positions of the 3D-printed IBT group.

Tooth number	Mesio-distal (0.5 mm)			Occluso-gingival (0.5 mm)			Bucco-lingual (0.5 mm)		
	Mean	SD	*P* value	Mean	SD	*P* value	Mean	SD	*P* value
Maxilla									
1	−0.01	0.16	0.57	−0.26	0.12	0.80	−0.02	0.18	**0.01**
2	0.00	0.1	0.76	−0.15	0.13	0.92	−0.01	0.14	**0.01**
3	0.00	0.25	0.96	−0.10	0.15	0.09	−0.16	0.23	0.06
4	0.02	0.12	0.96	0.08	0.18	0.14	−0.12	0.24	0.39
5	0.04	0.2	0.65	0.09	0.22	0.39	−0.06	0.27	0.24
6	0.02	0.07	0.34	0.03	0.05	0.59	0.01	0.06	0.08
Mandible									
1	−0.26	0.12	0.51	−0.29	0.15	0.88	−0.01	0.24	**0.01**
2	−0.15	0.13	0.72	−0.24	0.11	0.96	0.01	0.22	**0.01**
3	−0.10	0.15	0.39	−0.12	0.22	0.80	−0.01	0.26	0.07
4	0.08	0.18	0.33	−0.02	0.19	0.45	0.01	0.23	0.54
5	0.09	0.22	0.58	−0.06	0.18	0.08	0.11	0.22	0.28
6	0.03	0.05	0.72	0.02	0.07	**0.01**	0.06	0.07	0.26

Bold values signify the significant value set at *P* < 0.05.

**Table 5:** Difference of angular orientation between virtual and actual bracket position of the 3D-printed IBT group.

Tooth number	Torque (2°)			Rotation (2°)			Angulation (2°)		
	Mean	SD	*P* value	Mean	SD	*P* value	Mean	SD	*P* value
Maxilla									
1	−0.11	0.94	0.61	0.21	0.75	0.44	0.25	0.65	0.67
2	0.51	0.53	**0.02**	−0.33	1.03	0.24	0.21	0.52	0.57
3	0.11	0.66	0.05	0.04	0.17	0.31	0.14	0.63	0.61
4	0.02	0.17	0.14	0.11	0.41	0.24	0.00	0.14	0.72
5	−0.04	0.45	0.44	0.19	0.37	0.92	0.02	0.42	0.72
6	−0.20	0.47	0.05	−0.02	0.37	0.78	0.04	0.40	0.40
Mandible									
1	−0.06	0.14	0.28	−0.06	0.11	0.20	−0.18	1.22	0.77
2	0.10	0.39	0.86	−0.17	0.40	0.29	0.49	1.39	0.31
3	0.16	0.52	0.48	−0.05	0.56	0.65	0.25	0.93	0.58
4	0.33	0.57	0.08	0.19	0.36	0.19	0.03	0.42	0.91
5	0.22	0.36	0.10	−0.05	0.41	1.00	−0.09	0.24	0.41
6	0.10	1.00	0.54	0.01	0.46	0.55	0.15	0.74	0.68

Bold values signify the significant value set at *P* < 0.05.

**Table 6:** The difference of linear deviation between virtual and actual bracket positions of the double-layer vacuum-formed thermoplastic IBT group.

Tooth number	Mesio-distal (0.5 mm)			Occluso-gingival (0.5 mm)			Bucco-lingual (0.5 mm)		
	Mean	SD	*P* value	Mean	SD	*P* value	Mean	SD	*P* value
Maxilla									
1	0.06	0.09	0.08	−0.12	0.16	**0.04**	−0.09	0.12	0.06
2	0.08	0.10	**0.04**	−0.03	0.23	0.88	0.00	0.13	0.72
3	0.13	0.20	0.09	−0.06	0.37	0.51	0.04	0.17	0.88
4	0.29	0.15	**0.01**	−0.04	0.22	0.88	0.03	0.19	0.57
5	0.11	0.22	0.17	−0.09	0.19	0.57	−0.02	0.13	0.20
6	0.05	0.23	0.41	−0.08	0.21	**0.04**	−0.10	0.15	0.20
Mandible									
1	0.04	0.16	0.41	−0.01	0.27	0.26	−0.04	0.14	0.80
2	0.07	0.25	0.28	0.17	0.19	0.44	−0.01	0.13	**0.02**
3	0.18	0.16	**0.01**	0.05	0.15	0.26	−0.09	0.24	0.17
4	−0.04	0.18	0.65	0.19	0.16	0.20	−0.06	0.22	**0.01**
5	−0.02	0.25	0.96	0.27	0.23	0.51	−0.06	0.22	**0.01**
6	0.08	0.23	0.28	0.13	0.30	0.80	−0.06	0.27	**0.04**

Bold values signify the significant value set at *P* < 0.05.

**Table 7:** The difference in angular orientation between virtual and actual bracket positions of the double-layer vacuum-formed thermoplastic IBT group.

Tooth number	Torque (2°)			Rotation (2°)			Angulation (2°)		
	Mean	SD	*P* value	Mean	SD	*P* value	Mean	SD	*P* value
Maxilla									
1	−0.26	1.01	0.39	0.09	0.22	0.28	0.00	0.47	0.96
2	−0.34	1.10	0.33	−0.16	0.89	0.54	0.07	0.54	0.28
3	−0.10	0.79	0.80	0.06	0.48	0.96	0.44	0.38	**0.01**
4	−0.23	0.37	0.09	−0.09	0.89	0.59	0.12	0.31	0.54
5	0.12	0.80	0.54	0.43	0.70	0.14	0.09	0.79	0.39
6	−0.12	0.56	0.65	0.18	0.55	0.44	−0.20	0.46	0.12
Mandible									
1	0.06	0.46	0.76	−0.16	0.65	0.48	0.09	0.39	0.96
2	0.10	0.58	0.96	0.56	0.76	0.09	−0.05	0.57	0.39
3	−0.35	0.44	**0.01**	−0.16	0.45	0.51	0.23	0.38	0.17
4	0.13	0.64	0.44	−0.30	0.63	0.20	−0.01	0.36	0.65
5	0.04	0.70	0.76	−0.19	0.49	0.24	0.01	0.66	0.80
6	−0.21	0.33	0.17	−0.07	0.39	0.68	0.03	0.16	0.78

Bold values signify the significant value set at *P* < 0.05.

**Table 8:** Frequencies of bond failure during the removal of IBT in all groups.

Tooth no.		Insignia jig		3D-printed IBT		Double-layer IBT	
		U	L	U	L	U	L
1		0	0	0	2	0	3
2		0	1	0	1	0	2
3		0	0	0	0	0	0
4		1	1	1	0	1	2
5		1	2	0	1	1	3
6		1	0	0	0	0	0
Total	240	7 = 2.92%		5 = 2.08%		12 = 5.00%	
U and L							

## Data Availability

All the data supporting the results can be found under request through the correspondent's email at any time.

## Abbreviations

IBT: Indirect bonding trays
CX: Geomagic® control X™ software
STL: Stereolithography
3D: Three-dimensional
M-D: Mesio-distal
O-G: Occluso-gingival
B-L: Bucco-lingual
BCT: Buccal crown torque
LCT: Lingual crown torque
M-L: Mesio-lingual
M-B: Mesio-buccal
MCT: Mesial crown tip
DCT: Distal crown tip.

## Additional Points

*Limitations*. This study has several shortcomings, most significantly its small sample size. Customized orthodontic treatment is considerably more expensive than conventional one; we could not obtain a larger sample of patients due to socioeconomic reasons. The other shortcome, the current study was designed to limit confounding variables and thus used a single-operator protocol for the indirect bonding procedures. While this eliminates interoperator variability, it also reduces the generalizability of the results. On the other hand, this study targeted adults with Cl I malocclusions with mild to moderate crowding and treated without extraction. While such inclusivity aimed to mirror clinical practice, it could have also introduced variability in tray seating, adaptation, and ease of removal, all of which could influence bracket position. Since 2nd molars present challenges in both direct and indirect bonding, this study did not assess the indirect bonding of 2nd molars to reduce variability stemming from isolation and visibility. *Trial Registration*. This clinical trial was registered in ClinicalTrials.gov (NCT05549089) (https://clinicaltrials.gov/search?term=NCT05549089).
